# Flow Measurements in a Blood-Perfused Collagen Vessel Using X-Ray Micro-Particle Image Velocimetry

**DOI:** 10.1371/journal.pone.0081198

**Published:** 2013-11-18

**Authors:** Elizabeth Antoine, Cara Buchanan, Kamel Fezzaa, Wah-Keat Lee, M. Nichole Rylander, Pavlos Vlachos

**Affiliations:** 1 Department of Mechanical Engineering, Virginia Tech, Blacksburg, Virginia, United States of America; 2 VT-WFU School of Biomedical Engineering and Sciences, Virginia Tech, Blacksburg, Virginia, United States of America; 3 Advanced Photon Source, Argonne National Laboratory, Argonne, Illinois, United States of America; 4 School of Mechanical Engineering, Purdue University, West Lafayette, Indiana, United States of America; University of California, Irvine, United States of America

## Abstract

Blood-perfused tissue models are joining the emerging field of tumor engineering because they provide new avenues for modulation of the tumor microenvironment and preclinical evaluation of the therapeutic potential of new treatments. The characterization of fluid flow parameters in such *in-vitro* perfused tissue models is a critical step towards better understanding and manipulating the tumor microenvironment. However, traditional optical flow measurement methods are inapplicable because of the opacity of blood and the thickness of the tissue sample. In order to overcome the limitations of optical method we demonstrate the feasibility of using phase-contrast x-ray imaging to perform microscale particle image velocimetry (PIV) measurements of flow in blood perfused hydrated tissue-representative microvessels. However, phase contrast x-ray images significantly depart from the traditional PIV image paradigm, as they have high intensity background, very low signal-to-noise ratio, and volume integration effects. Hence, in order to achieve accurate measurements special attention must be paid to the image processing and PIV cross-correlation methodologies. Therefore we develop and demonstrate a methodology that incorporates image preprocessing as well as advanced PIV cross-correlation methods to result in measured velocities within experimental uncertainty.

## Introduction

Three-dimensional (3D) engineered microfluidic tissue models have recently emerged as valuable tools for the study of the progression of disease such as cancers, the development of therapeutics under controlled conditions, and regenerative medicine [1]–[9]. Three-dimensional tissue models are not only superior to two-dimensional cultures that poorly reflect the pathological microenvironment, but are also inexpensive and easily controlled in comparison to animal models and clinical trials [3], [8], [10]–[14]. Collagen I has become one of the most popular tissue models, as it forms the basis of natural extracellular matrix and can be easily formed into a 3D substrate for cell culture [15], [16]. Because fluid shear stress is known to play a pivotal role in several disease development processes such as tumor cell expansion, angiogenesis and metastasis [6], [17] as well as hemolysis and thrombosis formation [18], microfluidic vascular models are superior to static cultures. Characterization of the flow within perfused bioreactors in order to quantify fluid shear stress is critical towards understanding fluid-cell interactions [9], [19]. Particle image velocimetry (PIV) is a well-established noninvasive optical flow measurement technique that has been successfully used within various biological systems [20]–[22]. Our group has recently developed a novel *in-vitro* 3D perfused collagen-based tissue model for the study of cancer development and metastasis and successfully measured flow in this platform using microscopic PIV (µPIV); our initial studies have provided new insights into tumor-endothelial crosstalk under dynamic conditions [23].

A key challenge in tissue engineering is the transition from simple devices to more physiologically representative platforms. For example, vascular tumor models should be perfused with whole blood rather than cell culture media to better reproduce biological conditions of the *in-vivo* vasculature. However, because conventional PIV requires optical access to the region of interest, its use is limited to thin-walled transparent tissues perfused with a transparent fluid. Lima et al. have produced groundbreaking work in the development of μPIV techniques for blood flow measurement using a spinning-disk confocal PIV system [24]–[28]. Although their method has provided measurements at higher hematocrit and flow rates than were previously achieved, it is still restricted to measurement of flow in non-tissue transparent materials (PDMS or glass capillaries) with narrow channels (less than 100 μm along the optical axis) and sub-physiological flow rates (Re<0.025). Therefore the implementation of μPIV in whole blood-perfused cellularized thick tissues necessitates the development of a new methodology for μPIV.

Phase-contrast x-ray PIV is a recently developed variation of standard optical PIV that permits measurement of flow within opaque vessels [29]–[32]. In contrast to other technologies for flow measurement in opaque systems, such as MRI and nuclear imaging, x-ray PIV has high spatial and temporal resolution [33]. For conventional PIV measurements, tracer particles seeded within the flow of interest are illuminated by laser light and imaged with a high-speed camera. Subsequently, image pairs are correlated to calculate a velocity field with high spatio-temporal resolution [34], [35]. X-ray PIV replaces optical imaging with phase contrast imaging of x-ray-illuminated particles in the fluid volume. As such, the method is well-suited to characterization of *in vitro* and *in vivo* biological flows and a limited number of initial efforts have demonstrated PIV measurement in blood flows [36]–[39].

A significant limitation of phase-contrast x-ray imaging is that it provides extremely low signal to noise ratio images that are generally inadequate for PIV cross-correlation. Several groups have developed complex techniques to improve the quality of x-ray PIV, including optimization of experimental conditions [39], [40], alternative image processing algorithms [41], and application of various correlation algorithms [38], [42]. However, the techniques previously published are application-specific. Furthermore, previous work in x-ray PIV has focused on thin-walled rigid vessel geometries as well as *in vivo* measurements in very small microvessels near the tissue surface [33], [37]–[39]. It is hypothesized that that quality of phase-contrast x-ray images obtained in thick-walled tissues or tissue models will be still further decreased and require additional consideration for PIV processing. To our knowledge, no data have yet been published demonstrating successful measurement of blood flow in a thick-walled perfused tissue representative such as collagen.

This work demonstrates the feasibility of accurate x-ray phase contrast PIV in a thick tissue model and contrasts with data acquired in thin-walled rigid vessels. Experimental data is acquired in a blood-perfused collagen hydrogel, and a methodology is presented for PIV analysis of the resulting low SNR images using advanced methods. These include image preprocessing for enhancement of the signal-to-noise ratio as well as the use and advanced phase correlation approach to obtain more accurate velocity measurements. The following sections include descriptions of the experimental procedure used to obtain x-ray phase contrast images, discussion of advanced image processing and correlation techniques that are used to improve the PIV measurements, presentation of an optimal methodology, and evaluation of the results and performance of these methods.

## Materials and Methods

### Microvasculature models

Three sets of experimental conditions with varying experimental complexity were considered. The most basic condition was a rigid polymer polytetrafluoroethylene (PTFE) channel (756 μm diameter) perfused with a glycerine solution. The second channel (867 μm diameter) was rigid fluorinated ethylene propylene (FEP) perfused with whole bovine blood. Finally, the most complex experiment used a collagen I vessel bioreactor (742 μm diameter) perfused with whole bovine blood. PTFE and FEP are similar semi-rigid polymers that are non-reactive with blood, and tubing made of these materials is readily available with dimensions similar to microvasculature. Collagen I was selected for this experiment because it comprises the majority of tissue extracellular matrix, can be easily extracted from rat tail tendon, and is widely used as a matrix substrate for *in vitro* engineered tissues [43]. The collagen hydrogel provides mechanical and chemical stability while supporting cellular growth, organization, and structural remodeling similar to that found *in vivo* [15], [16].

**Figure 1:**
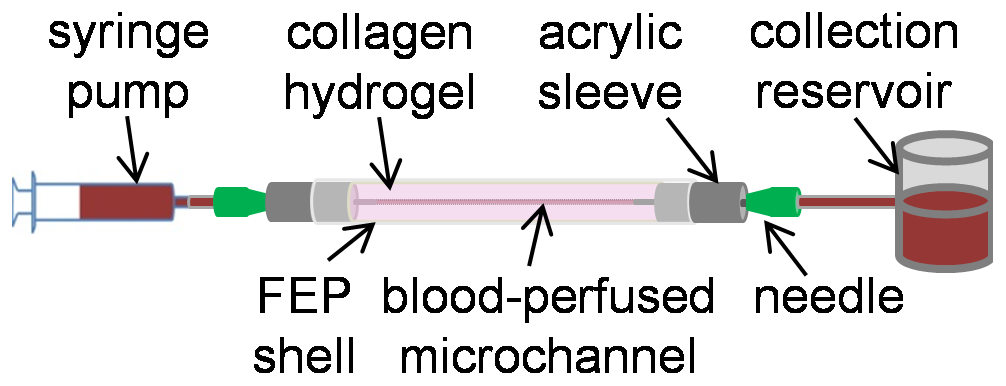
Diagram of blood-perfused collagen vessel bioreactor.

The collagen vessel was fabricated as described previously [23]. Type I collagen stock solution was prepared from rat tail tendons dissolved in pH-2.0 HCl and combined with 10× Dulbecco's Modified Eagle Medium (DMEM), 1N NaOH and deionized water to prepare a solution with 10 mg/mL collagen. The collagen solution was injected into a 5 cm long, 3 mm diameter cylindrical FEP shell fitted concentrically with a 5 cm long 22 gauge (711 μm) stainless steel needle and sealed at the ends with acrylic sleeves. The solution was cured for 30 minutes at 37°C, after which a cylindrical microchannel was created in the collagen hydrogel by removal of the needle. 1.27 cm long stainless steel needles were inserted into the acrylic sleeves at the ends of the collagen vessel to provide luer interfaces for connection with the flow system. Figure 1 depicts the collagen microchannel (not to scale). The PTFE and FEP channels were perfused with the same flow system using identical needles inserted into the ends of 5 cm sections of tubing and sealed with silicon adhesive.

### Working fluids and flow condition

Two working solutions were used for these experiments. A 50% v/v solution of water and glycerine was perfused in the PTFE channel. This solution was chosen because it closely matches the viscosity and density of blood and could therefore be perfused at the same rate as blood to achieve the same Reynolds number for flow similarity. The matched density also permitted the same neutrally buoyant tracer particles to be used in both blood and the glycerine solution. Fresh whole bovine blood (Bovine Blood in Citrate Anticoagulant, Quad Five) was perfused in both the FEP and collagen channels. The difference in source species between hydrogel and fluid (rat vs. bovine) is not expected to significantly affect the results as the hydrogel is formed after denaturation and reconstitution of tendon. Experiments were performed within 48 hours of the time that blood was drawn, and all procedures involving blood were performed under sterile conditions and using equipment washed with phosphate buffer solution (PBS). Blood was kept refrigerated until shortly before experiments were performed, at which time it was heated to room temperature (22 C). The dynamic viscosity of whole blood was measured to be 8.15 cP ± 5% and the viscosity of the 50% v/v water/glycerine solution was measured to be 9.5 cP ± 5%. Measurements were taken at 22°C and a shear rate of 30 s^−1^ using a Brookfield DVII+ Pro cone-plate viscometer. Because bovine RBCs further do not form rouleaux as do human RBCs [44], and the addition of citrate anticoagulant further shear dependence of viscosity, blood viscosity was assumed to be shear-independent in this study. The density of whole blood (1.06 g/cm^3^) [45] is nearly that of water, while that of a 50% v/v water/glycerine solution at 22°C is slightly higher at 1.14 g/cm^3^ [46].

The fluids were seeded with ∼4% v/v neutrally buoyant hollow glass microspheres (Sphericel, Potters Industries) with 10 μm nominal diameter. Hollow glass particles were selected because the density interface between air and glass creates high contrast for x-ray imaging; furthermore, they are suitable flow tracers as they are neutrally buoyant at a diameter of 10 μm. Because the negative surface charge acquired by glass immersed in water can activate the platelets in whole blood and initiate coagulation, it was necessary to first treat the glass microspheres with blood plasma to provide a protective protein coating on the glass surfaces before making a suspension of whole blood and particles. Whole bovine blood was centrifuged and the plasma supernatant was extracted. 220 mg of dry neutrally buoyant particles were suspended in 1 mL of plasma and incubated for 24 hours at 37° C. Finally, 5 mL whole blood was added to the plasma solution for the final working solution: blood seeded with 37 mg/mL neutrally buoyant hollow glass microspheres. For the water/glycerine working fluid, the highly concentrated filtered particle solution was diluted with distilled water to approximately twice the concentration of the whole blood solution and mixed with an equal volume of glycerine to obtain the second final working solution: 50% v/v water/glycerine seeded with approximately 40 mg/mL neutrally buoyant hollow glass microspheres.

Flow was generated using a syringe pump (Harvard Apparatus, PhD). Prior to flow measurements, the vessel was primed with the working fluid and flushed for several seconds at 5 mL/min to clear adhered and aggregated particles from the vessel. During flow measurements, the fluid was perfused steadily through the vessel at a flow rate *Q* = 5 μL/min for a theoretical peak velocity of 0.3–0.4 mm/s depending on vessel diameter. The Reynolds number was approximately 0.03 for all experimental cases.

### Phase-contrast X-Ray Imaging

**Figure 2:**
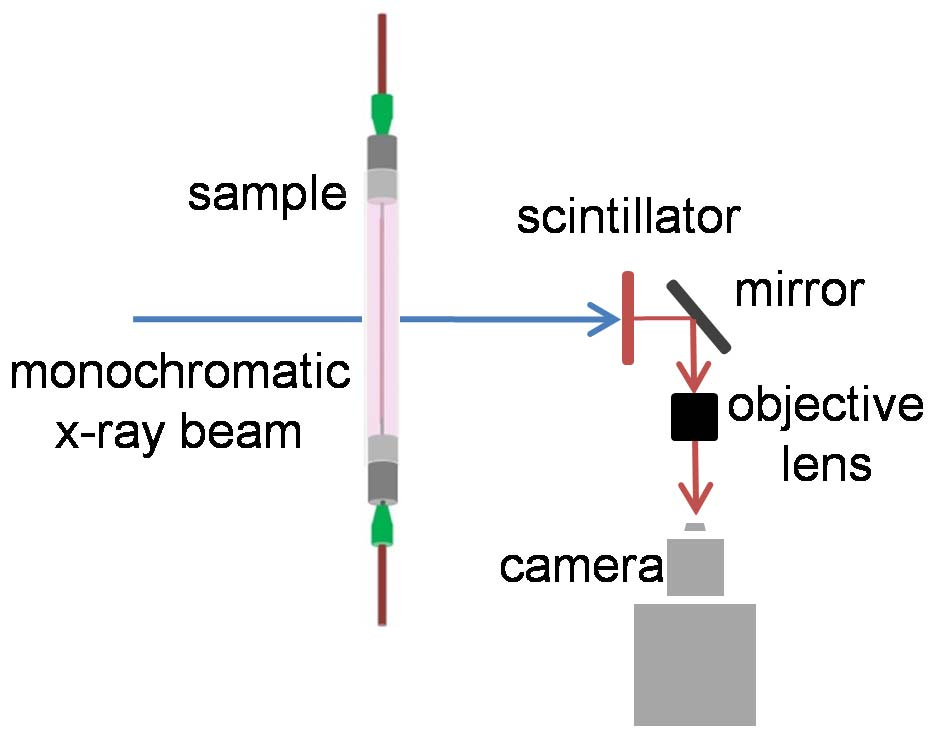
Schematic of x-ray PIV in collagen vessel bioreactor.

X-ray particle images were acquired at the X-ray Science Division beamline 32-ID of the Advanced Photon Source (APS) at Argonne National Laboratory. For each experiment, the vessel (PTFE, FEP, or collagen microvessel) was fixed in the path of a monochromatic x-ray beam with 1 mm^2^ cross-sectional area. The x-ray source was the standard APS undulator A, set to 15.5 mm gap. The energy of the beam, controlled by a cryogenically cooled double crystal monochromator (DCM) (Si 1 1 1) with energy band pass dE/E∼10^−4^, was 18 keV. The beam was converted into visible light by a 100 µm thick YAG:Ce scintillator crystal and magnified with a 5× lens (LWD objective, Mitutoyo) for imaging (Figure 2). 1.2 megapixel 8-bit images were recorded at *f* = 20 or 30 Hz (depending on the experimental case) with a machine-vision camera (AVT Pike F-505B). Images of a 400-mesh gold transmission electron microscopy (TEM) reference grid were recorded and used to measure the effective pixel size of 1.32 μm/pixel. A minimum of 2000 images was collected for each experimental case.

## Results

### Effect of Image Preprocessing on Image Signal-to-Noise Ratio and PIV Accuracy

**Figure 3:**
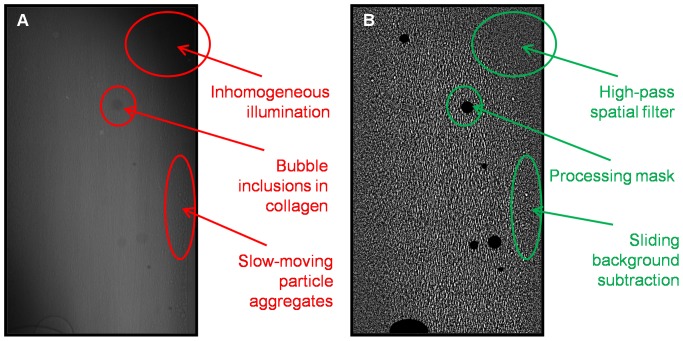
Sample images of blood flow in the collagen microchannel. A: Raw image after reorientation and cropping to vessel diameter. B: Processed image (sliding mean background subtraction, high-pass filter, and mask). Image B has been brightness- and contrast-enhanced for visualization.

While fluorescent particles used in optical μPIV are excited by and emit high intensity light, in phase-contrast x-ray imaging intensity variations are generated due to material density differences that result in changes in the index of refraction for propagating monochromatic x-ray beams. Particle images in optical PIV ideally have a Gaussian intensity distribution, while particle information in x-ray phase contrast images is manifested by interference patterns in the intensity distribution, such as a bright center and dark circumference for a hollow glass sphere [47]. Furthermore, phase-contrast x-ray images can exhibit high and non-uniform background intensity due to artifacts in the incident beam and image formation process, and experimental conditions can lead to the presence of particle aggregation and further artifacts, as shown by a representative image in Figure 3A. Hence, image pre-processing is essential.

Typical image processing involves background removal using subtraction of the mean image generated by averaging across the whole dataset [48]. For the data presented here, this procedure was modified and a temporal mean background subtraction [49] with a window of 10 images was performed. In temporal mean background subtraction, at each time point the mean image subtracted from the current frame is computed as the average of frames in a temporal window centered on the current frame. This processing technique was used to overcome particle aggregation effects: despite the plasma treatment, glass flow tracers form aggregates that roll slowly along the walls of the channel, not following the flow and biasing the velocity measurements. The particle aggregates move sufficiently during recording of a data set (>2000 frames) that they do not contribute to the mean of the entire data set and are retained in traditional background subtraction; however, they remain stationary in a 10-image mean and can thus be effectively removed. In contrast, flow-tracing particles, even those near the vessel walls, move sufficiently during the 10-frame window that they are retained after background subtraction and their signal is not lost.

Subsequently, a high-pass frequency-domain Gaussian filter was selected to equalize illumination across the image, revealing intensity fluctuations due to the flow tracers or other density variations. Finally, the process of manufacturing the collagen microchannels produces unavoidable micro-bubbles in the hydrogel. Given the high density difference between the collagen and the micro-bubbles and the volume integration nature of x-ray image recording, such artifacts can completely obscure the flow. This was addressed by applying a binary mask to the image so that correlations would not be performed in the regions where the image is known to have no meaningful information. Here, the mask was manually drawn over collagen inclusions based on visual inspection of the raw images.

The effect of image processing is shown in Figure 3B. In comparison with the raw image in Figure 3A, individual particles can be visually identified in the processed image and, although contrast enhancement is necessary for visualization for Figure 3B, it is clear that the contrast between particles and background is significantly higher. Furthermore, near-wall particle aggregates have been effectively removed and low-frequency intensity variation across the image has been successfully eliminated. Masked regions are shown in solid black in Figure 3B. From Figure 3B however, it is evident that the resulting images are more representative of a speckle pattern. Furthermore, the particles exhibit a degree of longitudinal "smearing" due to their displacement during the camera exposure. The images therefore do not resemble conventional PIV images.

**Figure 4:**
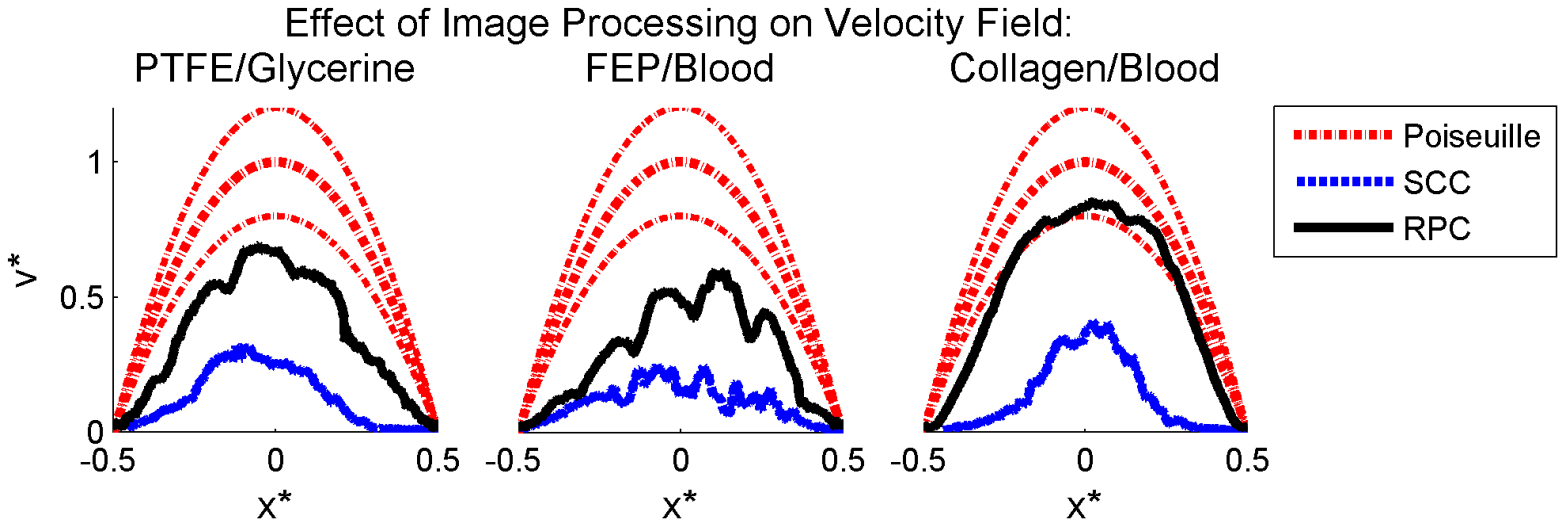
Effect of image preprocessing on PIV accuracy. Mean velocity profiles obtained using standard PIV correlation applied to raw images (dashed blue lines) and processed images (solid black lines) are plotted with the volume-adjusted Poiseuille solution (dashed red lines) for each experimental case. The uncertainty based on measured flow rate and vessel diameter is plotted for the theoretical solution (upper and lower dashed red lines). The 95% confidence interval on the mean for the experimental data is not distinguishable from the mean. Left to right: glycerine-perfused PTFE, blood-perfused FEP, and blood-perfused collagen. Data shown is averaged in time and along the length of the vessel and is normalized for each experimental case by the vessel diameter and theoretical maximum velocity.

To determine the effect of image processing on velocity field accuracy, PIV was performed on both raw and processed images. Correlation windows of 4×64 pixels with 75% overlap were selected to satisfy the optimization conditions published in [50] for the case of uniaxial high-shear flow. Single-pass standard cross-correlation (SCC) with zero-mean windows and three-point Gaussian (3PG) peak detection was applied and correlations were validated using a median universal outlier detection (UOD) scheme [51] with a 9×9 stencil and velocity thresholding. Secondary peaks were interrogated as potential replacements for invalid vectors. Instantaneous vector fields were averaged in time for post-processing; the results are shown in Figure 4. The volume-adjusted velocity profile used as a reference for comparison with the volumetric PIV data is computed by integration of the axisymmetric Poiseuille solution along the x-ray beam path through the channel. This results in a parabolic profile with two-thirds the magnitude of the centerline velocity profile [32]. For all three experimental cases, the unprocessed images result in severe underestimation of the velocity field, with RMS deviation of the velocity profile from the true value near 60% for all cases. Image processing significantly increases the accuracy of the velocity field, resulting in vector fields with only 10–30% RMS deviation from the true value. Although this error is still significant and further improvement of data processing is necessary, this result demonstrates that basic image processing alone can reduce the error in mean PIV velocity fields by over 50%.

The improvement in correlation accuracy after image processing is expected to be due to removal of the non-moving background and artifacts, or “noise”, which were correlating far more strongly than the weak signal provided by the particles. Removal of this background, artifacts, and error-generating intensity fluctuations should have increased the signal-to-noise ratio, or SNR, resulting in correlation peaks closer to the true particle displacement. To determine quantitatively the effect of image processing, the SNR of each image set was examined before and after image processing.

In microPIV applications, SNR has previously been calculated as the ratio of the peak intensity of a typical particle to the mean background intensity [52]. This definition not only assumes that the particle images have a Gaussian distribution, but also requires that individual particles be readily identified. Neither of these conditions is met by x-ray phase contrast images, requiring the use of an alternative definition of SNR. Here we calculate SNR using the peak-to-root mean square ratio (PRMSR) as described by Kumar et al. [53], where the signal component is represented by the magnitude of the image autocorrelation peak *y_0_* and noise component is the RMS of values outside the correlation peak *y_RMS_* (Equation 1). Here, following Kumar et al., the threshold to separate the peak from the noise is chosen to be 50% of the peak magnitude. This SNR provides an estimate of image quality with no dependence on external experimental parameters or assumptions about image properties.



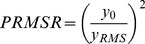
(Equation)


**Figure 5:**
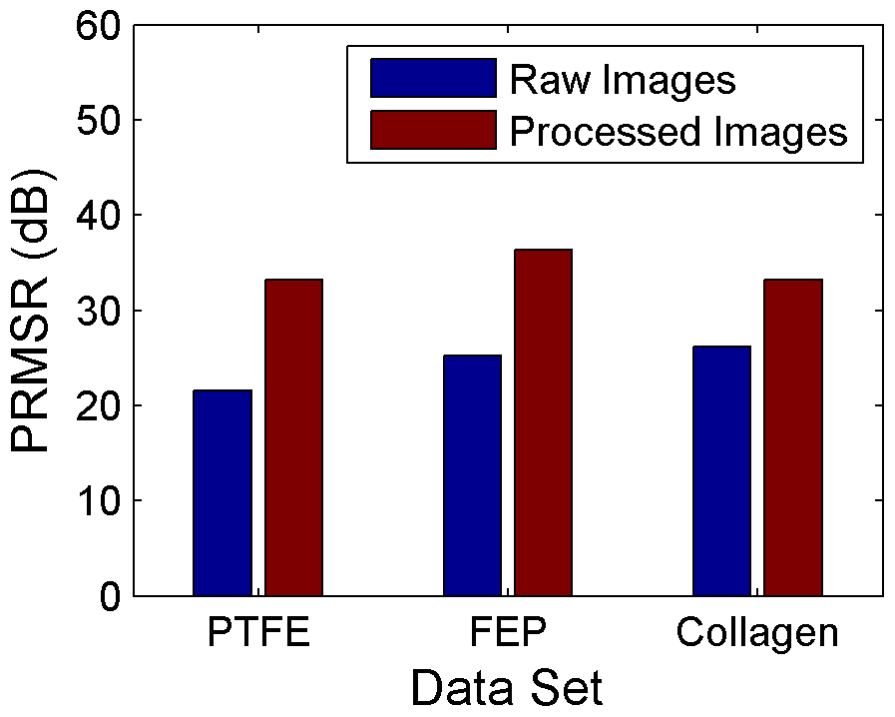
Comparison of image signal-to-noise ratio before and after image preprocessing. The mean SNR of all images (>2000) is plotted for each data set.

The PRMSR was calculated for 32×32 pixel blocks of each image and the median value of all blocks was taken as the SNR of each image. Finally, the SNR of each experimental case was computed as the mean SNR of all images in the data set, for both the raw and processed images. The results, shown in Figure 5, confirm the hypothesis that the image processing significantly improved image quality: the SNR for the processed images is at least 7 dB higher than the SNR for the raw images. This higher SNR for the processed images corresponds to the improved accuracy of the corresponding velocity fields in Figure 4, indicating that this definition of SNR can be a good predictor of relative correlation accuracy even though it contains only single-image intensity information.

### Effect of Robust Phase Correlation on PIV Accuracy

The benefit of image processing to improve SNR for low-quality images has been established; however, even improved images give inaccurate results using SCC. This is because the images still contain significant noise levels, and the correlation peaks are too broad for accurate peak detection. Eckstein et al. developed an advanced correlation technique known as robust phase correlation (RPC) which applies an SNR filter to the image cross-correlation [54], [55]. The SNR filter is a spectral filter weighted based on a theoretical SNR, which assumes that the signal is composed of Gaussian particles with a specified input diameter, here defined as 2.8 pixels. Typically RPC provides a sharper peak with better peak detectability than SCC and results in higher accuracy for velocity measurements.

**Figure 6:**
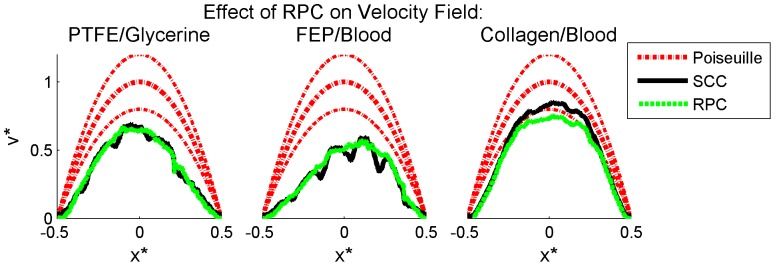
Effect of PIV correlation method on PIV accuracy. Mean velocity profiles obtained using standard cross-correlation (solid black lines) and robust phase correlation with 2.8 pixel diameter (dashed green lines) applied to processed images are plotted with the volume-adjusted Poiseuille solution (dashed red lines) for each experimental case. The uncertainty based on measured flow rate and vessel diameter is plotted for the theoretical solution (upper and lower dashed red lines). The 95% confidence interval on the mean for the experimental data is not distinguishable from the mean. Left to right: glycerine-perfused PTFE, blood-perfused FEP, and blood-perfused collagen. Data shown is averaged in time and along the length of the vessel and is normalized for each experimental case by the vessel diameter and theoretical maximum velocity.

PIV correlations are typically performed in the Fourier domain to improve computational efficiency; however, the application of Fourier transforms to discrete data introduces error. A 50% Gaussian windowing filter, which has been shown by Eckstein et al. to increase measurement resolution and accuracy by minimizing this error [56], was applied in the Fourier domain to 8×128 pixel regions to obtain post-filter correlation windows of 4×64 pixels, the same dimensions as used for SCC processing. Single-pass RPC with 2.8 pixel particle diameter was applied using the same zero-mean windows, 3PG peak detection, and validation scheme as used for SCC processing, and the resulting vector fields were subsequently time-averaged. Figure 6 shows both the original SCC and the new RPC velocity profiles. For all three experimental cases, the application of RPC and the windowing filter has a smoothing effect on the velocity profile but has little effect on the magnitude of the velocity profile. In fact, for the case of blood-perfused collagen the addition of RPC and windowing negatively biased the resulting velocity field.

**Figure 7:**
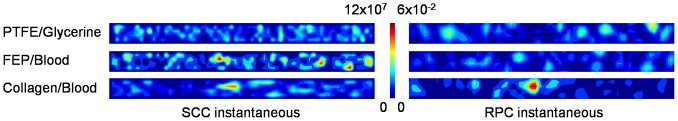
Contours of SCC and RPC correlation planes. The contour indicates relative correlation magnitude. Only the central 50% window is shown for RPC correlation planes.

To examine the effect of RPC on correlation accuracy, SCC and RPC correlation planes from a representative correlation region positioned midway between the centerline and wall were plotted for each experimental case (Figure 7). Although RPC provides sharper peaks in the correlation plane, multiple peaks of similar magnitude can still be present. This indicates that while subpixel accuracy is improved for RPC over SCC, the peak corresponding to the correct displacement may not be accurately determined.

### Sum-of-Correlation for Improved Correlation Accuracy

As demonstrated in the previous section, even with the use of RPC, multiple peaks remain in the correlation planes, leading to invalid vector detection and contaminating the mean velocity profiles. Sum-of-correlation, also known as ensemble correlation, is a technique which can be applied to steady or periodic flows and is performed by averaging instantaneous correlation functions prior to peak detection rather than after estimation of velocity fields [57], [58]. This technique increases correlation accuracy and resolution by effectively increasing the signal in each correlation, and has been used to enable accurate measurements in flows with low seeding and/or low SNR. Because of this, sum-of-correlation is ideally suited to this experimental data.

**Figure 8:**
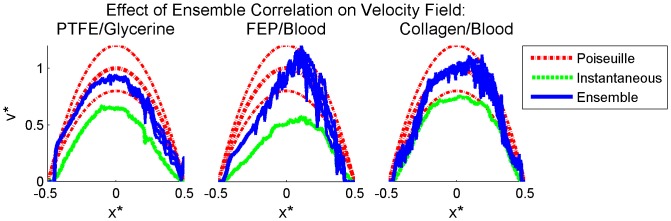
Effect of sum-of-correlation on PIV accuracy. Mean velocity profiles obtained using instantaneous (dashed green lines) and sum-of-correlation (solid blue lines) RPC applied to processed images are plotted with the volume-adjusted Poiseuille solution (dashed red lines) for each experimental case. The uncertainty based on measured flow rate and vessel diameter is plotted for the theoretical solution (upper and lower dashed red lines). The 95% confidence interval on the mean for the experimental data is not distinguishable from the mean. Left to right: glycerine-perfused PTFE, blood-perfused FEP, and blood-perfused collagen. Data shown is averaged in time for the instantaneous sets and along the length of the vessel for all sets and is normalized for each experimental case by the vessel diameter and theoretical maximum velocity.

Image pairs were correlated using a single-pass RPC sum-of-correlation algorithm. All other parameters were identical to the previous processing algorithm, including 50% Gaussian windowing, window dimensions, RPC diameter, zero-mean windows and 3PG peak detection. Validation was again performed using a 9×9 UOD scheme and thresholding with secondary peak replacement. Because sum-of-correlation uses the temporal component of the data in the correlation, a single vector field resulted, which was subsequently averaged along the length of the vessel. Figure 8 compares instantaneous and sum-of-correlation velocity profiles. For all three experimental cases, sum-of-correlation increases the magnitude of the velocity profile, increasing its accuracy.

**Figure 9:**
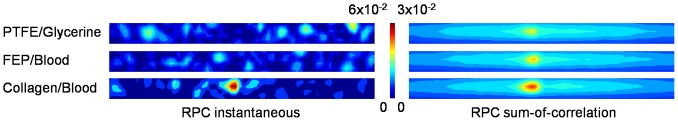
Contours of RPC instantaneous and sum-of-correlation planes. The contour indicates relative correlation magnitude. Only the central 50% window is shown.

As done previously to compare SCC and RPC, correlation planes were plotted for a representative correlation region positioned midway between the centerline and wall to examine the effect of sum-of-correlation on correlation accuracy (Figure 9). The effect of sum-of-correlation is clear: where multiple scattered peaks are present in the instantaneous correlation planes, the sum of all correlation planes at that location provides a single peak at the correct displacement, indicating that this technique is very effective at removing random noise.

## Discussion

The experimental error for all analyses, calculated as the RMS of the error in the experimentally measured velocity fields, is given in Table 1. These data quantify the improved accuracy obtained by each successive modification of the PIV algorithm. For all experimental cases and correlation schemes, advanced image preprocessing increases accuracy. For all experimental cases, the optimal PIV processing scheme includes not only image processing, but also RPC sum-of-correlation. For the glycerine-perfused PTFE and blood-perfused collagen cases, the resulting velocity field has less than 10% error, which is less than the 14.6% uncertainty of the theoretical solution. The FEP case deviates more strongly from the Poiseuille profile, with just over 17% error. For this case, there is significant asymmetry in the measured velocity profiles; this is most likely an experimental artifact due to slight curvature along the length of the vessel.

**Table 1:** RMS deviation of experimental mean velocity profile from volume-adjusted theoretical solution, normalized by volume-adjusted theoretical maximum displacement.

Experimental Case	Image Set	PIV Processing Scheme	RMS Error (normalized)
PTFE/glycerine	Raw	SCC instantaneous	0.5807
	Processed	SCC instantaneous	0.3092
		RPC instantaneous	0.3087
		RPC sum-of-correlation	0.0966*
FEP/blood	Raw	SCC instantaneous	0.6062
	Processed	SCC instantaneous	0.3989
		RPC instantaneous	0.3836
		RPC sum-of-correlation	0.1732
Collagen/blood	Raw	SCC instantaneous	0.5719
	Processed	SCC instantaneous	0.1576
		RPC instantaneous	0.2077
		RPC sum-of-correlation	0.0890*

* These values are within the uncertainty of the theoretical solution. The RMS uncertainty of the theoretical Poiseuille solution was computed by propagation of error obtained from manufacturer-provided uncertainties of the pump flow rate and tubing diameter and was determined to be 0.1461 (normalized by the volume-adjusted theoretical maximum displacement).

### Limitations and Future Directions

Due to limited access to the APS facility, multiple runs of these experiments were not feasible. In particular, this limits the information that can be derived from the FEP case because of the channel asymmetry. While the flow rate was lower than physiological in vessels of this diameter, this is not expected to detract from the results as it is still high enough to obtain shear-independent viscosity and Newtonian flow characteristics. Finally, it is clear from the data that it is not of sufficient fidelity for accurate near-wall flow measurements, but rather provides an overall picture of the velocity magnitude and distribution across the channel. Although the collagen hydrogel was not cellularized in these experiments, is it possible that their presence could contribute to image noise; however, as they would be stationary during the duration of experiments, this effect could be removed in the background subtraction stage of image processing.

It should be noted that comparison of experimental data with the Poiseuille solution integrated along the beam axis implicitly assumes that the experimental solution is derived from the geometric mean of velocities within the correlation window. However, as demonstrated by Fouras et al. [30], this is not strictly accurate as correlation peak detection returns a velocity which represents the mode rather than the mean. Optimization of PIV methods could be used to improve the velocity estimation. Similarly, the RPC filter, which was optimized for particles with Gaussian intensity distribution in optical PIV, could be modified for more effective application to x-ray phase contrast images. This would require assessment of x-ray phase contrast image formation for development of a new SNR model to be used in the filter.

## Conclusions

This work advances previous x-ray PIV technology by demonstrating for the first time flow measurement in an opaque blood-perfused *in vitro* microvascular engineered tissue. We found that the use of a thick, hydrated collagen tissue model has no effect on the quality of the phase-contrast x-ray images, which is promising for future work in complex cellularized tissue models. The methodology presented here outlines an approach for the design of PIV analysis to obtain high-quality flow measurements for challenging data, paving the way toward future experimental investigation of *in vitro* tissue vascular flow.
